# Association between organochlorine pesticides and nonalcoholic fatty liver disease in the National Health and Nutrition Examination Survey 2003–2004

**DOI:** 10.1038/s41598-022-15741-2

**Published:** 2022-07-08

**Authors:** Hyunji Sang, Kyu-Na Lee, Chang Hee Jung, Kyungdo Han, Eun Hee Koh

**Affiliations:** 1 Department of Internal Medicine, Asan Medical Center, University of Ulsan College of Medicine, Seoul, Republic of Korea; 2 Department of Biomedicine & Health Science, The Catholic University of Korea, Seoul, Republic of Korea; 3 Department of Statistics and Actuarial Science, Soongsil University, Seoul, Republic of Korea

**Keywords:** Endocrine system and metabolic diseases, Non-alcoholic fatty liver disease, Risk factors, Epidemiology, Environmental impact

## Abstract

While endocrine disruptors are emerging as a cause of nonalcoholic fatty liver disease (NAFLD), little is known about the link between NAFLD and organochlorine pesticides (OCPs), one of the endocrine disruptors. We retrospectively analyzed the U.S. National Health and Nutrition Examination Survey 2003–2004 and compared the baseline demographics in individuals according to the presence of NAFLD (fatty liver index [FLI] ≥ 60). Logistic regression analysis was performed to determine whether OCP concentration affected NAFLD prevalence and subgroup analyses regarding NAFLD-related variables and advanced hepatic fibrosis (FIB-4 ≥ 2.67) were performed. Of the 1515 individuals, 579 (38.2%) had NAFLD. Oxychlordane showed concentration-dependent risk for NAFLD (OR 3.471 in fourth quartile [Q4]; 95% CI 1.865–6.458; *P* = 0.007). *p,p′*-DDE and trans-nonachlor showed similar trends without statistical significance. Conversely, mirex showed the lowest risk for NAFLD in the highest concentration quartile (OR 0.29 in Q4; 95% CI 0.175–0.483; *P* < 0.001). Oxychlordane showed the most pronounced association with the levels of each component of FLI and liver enzymes. None of the OCPs were significantly associated with advanced fibrosis. In conclusion, among OCPs, exposure to oxychlordane showed the most prominent impact associated with NAFLD.

## Introduction

Endocrine-disrupting chemicals (EDCs) disrupt various metabolisms and hormonal signaling pathways involved in homeostasis^1^. EDCs are primarily synthetic and found in multiple substances such as pesticides, metals, additives, contaminants in food, and personal care products. As such, humans are exposed to EDCs through food ingestion, respiratory inhalation, and skin contact.

Among EDCs, organochlorine pesticides (OCPs) were widely used in agriculture in the 1940s. OCPs have high persistence, low polarity, low aqueous solubility, and high lipid solubility^2^. The half-life of OCPs varies from 60 days to 10–15 years^3^. Even after the Stockholm Convention banned their use internationally, OCPs have remained in the environment and accumulated through the food chain^4^. As such, they can enter the human body through long-term consumption of foods with high fat content^1^.

Exposure to OCPs is clinically important due to their association with obesity, insulin resistance, type 2 diabetes, dyslipidemia, and elevated liver enzyme levels^5–11^. Accordingly, these factors are also related to nonalcoholic fatty liver disease (NAFLD), which encompasses a wide range of pathologies from simple hepatic steatosis to nonalcoholic steatohepatitis and can progress to cirrhosis^12,13^. With the recent increase in the prevalence of NAFLD in Western countries, EDCs have emerged as one of the promising possibilities, and hidden environmental factors such as OCPs have been suspected as causative factors^14^. However, only few studies have evaluated the association between OCPs and NAFLD using population-based data^15^.

This study aimed to determine the association between OCP exposure and NAFLD as determined using fatty liver index (FLI). We also investigated the relationship between OCP exposure and other NAFLD-related variables, including liver enzymes and advanced hepatic fibrosis.

## Methods

### Data source and study population

This was a cross-sectional study based on the National Health and Nutrition Examination Survey (NHANES) 2003–2004 census in the United States. The Centers for Disease Control and Prevention, in conjunction with NHANES, uses biomonitoring to provide ongoing assessments of the US population’s exposure to environmental chemicals. The National Center for Health Statistics Research Ethics Review Board approved the collection of the NHANES 2003–2004 data. Analysis of de-identified survey data is exempt from federal regulations for the protection of human research participants. All methods for this NHANES study were performed in accordance with the relevant guidelines and regulations. Accordingly, the Asan Medical Center Institutional Review Board (AMC IRB) reviewed the protocol of this study and exempted it from review because it included only secondary analyses of de-identified data (IRB number: 2021–1570).

Adults aged 20 years or older were included in the analysis, and patients positive for hepatitis B or C virus and heavy drinkers were excluded. Heavy drinkers were defined as men who consumed more than 30 g of alcohol per day and women who consumed more than 20 g of alcohol per day^16^.

### Organochlorine pesticides (OCPs)

The subclasses of OCPs were measured from serum samples using high-resolution gas chromatography/isotope-dilution high-resolution mass spectrometry (HRGC/ID-HRMS). Serum levels of chlordane metabolites, including oxychlordane and trans-nonachlor, are still significant in the US population^17^. We therefore selected the following subclasses of OCPs for analysis: *p,p′*-dichlorodiphenyldichloroethylene (DDE), oxychlordane, trans-nonachlor, and mirex. The serum concentrations of other OCP subclasses such as hexachlorobenzene, heptachlor epoxide, aldrin, dieldrin, and endrin were below the detection limit and excluded because meaningful results could not be derived thereof.

### Outcome variables

We used the following data from NHANES 2003–2004 data: sex, race, smoking status, alcohol use behavior, intensity and frequency of physical exercise, income, age, height, body weight, waist circumference, serum creatinine, fasting serum glucose, total cholesterol, triglyceride, AST, ALT, GGT, hepatitis B surface antigen, and hepatitis C virus antibody. FLI was calculated using the following equation: FLI = (e^0.953 × loge (triglyceride) + 0.139 × BMI + 0.718 × loge (GGT) + 0.053 × waist circumference − 15.745^)/(1 + e^0.953 × loge (triglyceride) + 0.139 × BMI + 0.718 × loge (GGT) + 0.053 × waist circumference − 15.745^) × 100^18^. NAFLD was defined as FLI ≥ 60 and excluded if the FLI was less than 30. In a previous study, the sensitivity and specificity of FLI ≥ 60 for fatty liver is 61% and 86%, respectively^18^; accordingly, the presence of NAFLD was defined according to the FLI cutoff value of 60. As such, participants with serum OCP concentration measurements were divided into two groups based on the FLI cutoff value of 60.

The fibrosis-4 index (FIB-4), which is used to predict advanced hepatic fibrosis, consists of age, platelet, AST, and ALT and can be calculated relatively simply^19^. FIB-4 of 2.67 or higher can be used to predict advanced fibrosis in NAFLD^20^.

### Statistical analyses

Baseline characteristics of the study population were compared between those with NAFLD (FLI ≥ 60) and those without (FLI < 60) using Student’s *t*-test for continuous variables and the chi-squared test for categorical variables. The association between serum OCP concentration and the presence of NAFLD was analyzed. According to the cumulative exposure ranking, the concentration of each OCP in participants was also divided into quartiles (Q1 to Q4). The first quartile was defined as those exposed to the lowest concentrations, and the fourth quartile referred to those exposed to the highest concentrations. Supplementary Table S1 shows the cutoff values for each quartile when dividing the participants into the quartiles according to the cumulative exposure rankings for each OCP substance. We also analyzed the association between advanced hepatic fibrosis and OCPs by calculating the adjusted odds ratio (OR) for an FIB-4 score of 2.67 or higher according to the degree of OCP exposure.

The associations between each quartile of OCPs and cutoff values of FLI (i.e., ≥ 60 vs. < 60) and FIB-4 (i.e., ≥ 2.67 vs. < 2.67) were analyzed using logistic regression analysis. Three adjusted models were generated as follows: model 1, non-adjusted; model 2, adjusted for age, sex, and race; model 3, adjusted for age, sex, race, income, smoking, drinking, and physical activity. The adjusted OR for the presence of NAFLD (i.e., FLI ≥ 60) in the remaining quartile groups was calculated by setting the first quartile group as the reference. Pearson’s correlation analysis was performed to evaluate the correlation between serum OCP concentration and the variables constituting FLI (i.e., triglyceride, waist circumference, BMI, and GGT). Also, the adjusted means of other variables of NAFLD (e.g., AST, ALT, and GGT) were compared according to the quartiles using ANCOVA. For all analyses, statistical significance was defined at *P* < 0.05. SAS version 9.4 (SAS Institute Inc., Cary, NC, USA) was used for analysis.

## Results

### Demographic information

**Figure. 1:**
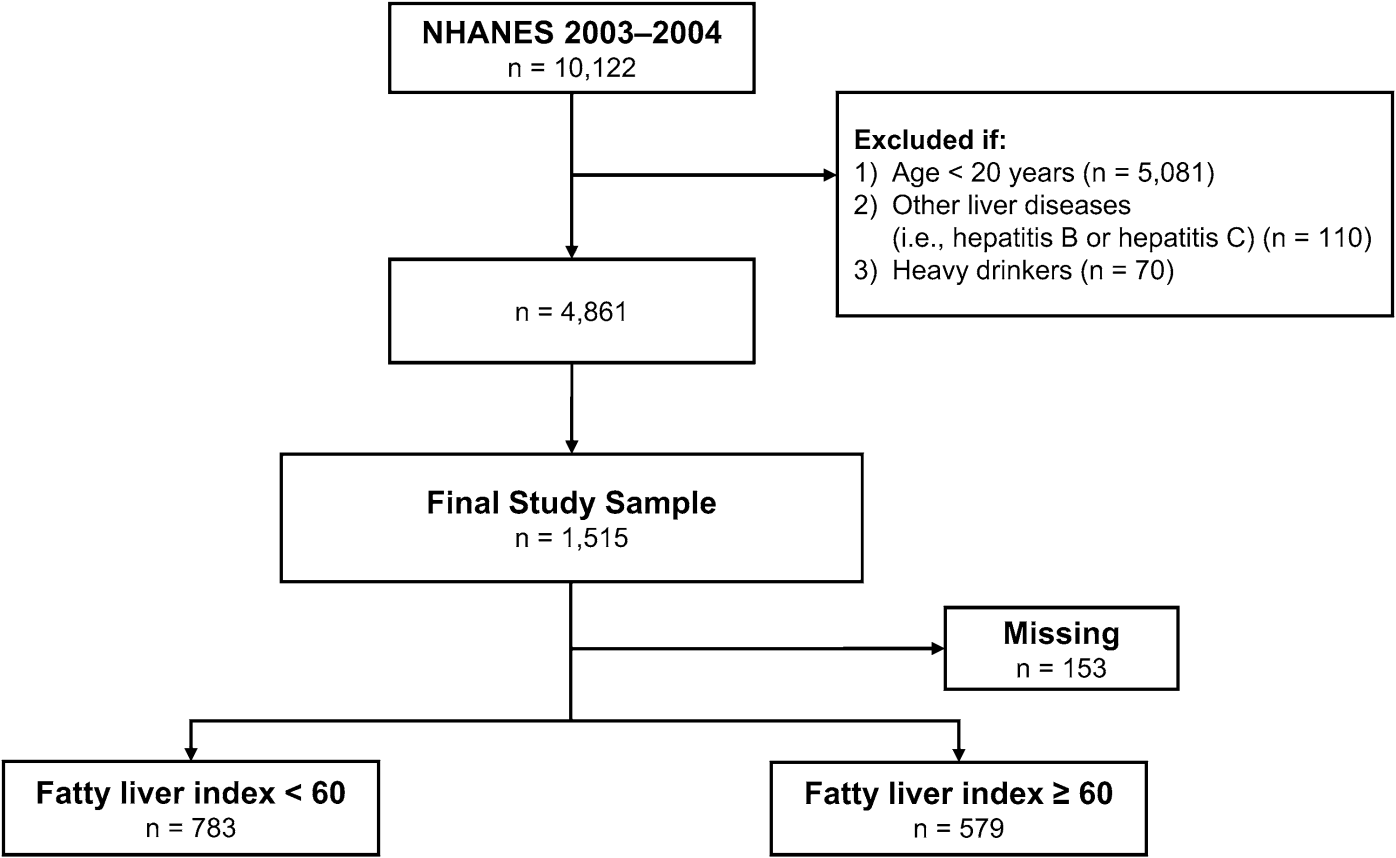
Study population selection flowchart.

A total of 4861 adults remained after applying the exclusion criteria. Of them, serum OCP concentrations were measured in 1515 participants, who were divided into two groups based on the FLI value cutoff of 60. There were 579 participants who had an FLI of 60 or higher and were therefore classified as having NAFLD (Fig. 1). Table 1 shows the clinical characteristics of the participants in the two groups. Compared with those without NAFLD, the NAFLD group was older and had higher proportions of male sex and those with obesity, low physical activity, hyperglycemia, hypercholesterolemia, hypertriglyceridemia, and high liver enzyme levels (AST, ALT, and GGT). There were no significant differences between the two groups in terms of race, smoking, alcohol drinking, and poverty income ratio.

**Table 1:** Clinical characteristics of the participants according to fatty liver index.

	Fatty liver index		*P* value
	< 60	≥ 60	
	n = 783	n = 579	
**Sex**			< 0.001
Male	39.1%	58.2%	
Female	60.9%	41.8%	
**Race**			0.49
Mexican American	7.3%	8.1%	
Other Hispanic	3.5%	3.8%	
Non-Hispanic White	72.8%	74.4%	
Non-Hispanic Black	9.9%	10.1%	
Other	6.5%	3.7%	
**Smoking**			0.14
Never	51.6%	45.7%	
Ex-smoker	22.8%	29.6%	
Current smoker	25.6%	24.7%	
Never drinker	11.3%	11.7%	0.83
Physical activity^a^	38.8%	26.5%	< 0.001
Low-income^b^	39.5%	35.3%	0.19
Age (years)	44.71 ± 0.77	47.94 ± 0.77	< 0.001
BMI (kg/m^2^)	24.47 ± 0.09	33.4 ± 0.39	< 0.001
Waist circumference (cm)	88.1 ± 0.43	110.96 ± 0.65	< 0.001
Creatinine (mg/dL)	0.87 ± 0.01	0.9 ± 0.01	0.003
Glucose (mg/dL)	94.7 ± 1.13	107.81 ± 2.04	< 0.001
Total cholesterol (mg/dL)	199.53 ± 1.88	210.97 ± 1.7	0.007
Triglyceride (mg/dL)	86.19 (81.87–90.74)	162.85 (151.97–174.51)	< 0.001
AST (mg/dL)	22.37 (21.75–23.02)	24.87 (24.27–25.48)	< 0.001
ALT (mg/dL)	19.89 (19.17–20.63)	26.76 (25.75–27.81)	< 0.001
GGT (mg/dL)	16.53 (15.56–17.55)	28.22 (26.71–29.82)	< 0.001

All categorical data presented as percent, and continuous data presented as mean (standard errors) or geometric mean (95% confidence intervals).

*AST* aspartate transaminase, *ALT* alanine transaminase, *GGT* gamma-glutamyl transferase.

^a^Defined as moderate or vigorous activity over the past 30 days.

^b^Poverty income ratio < 2.

### Relationship between OCPs and the presence of NAFLD

**Figure. 2:**
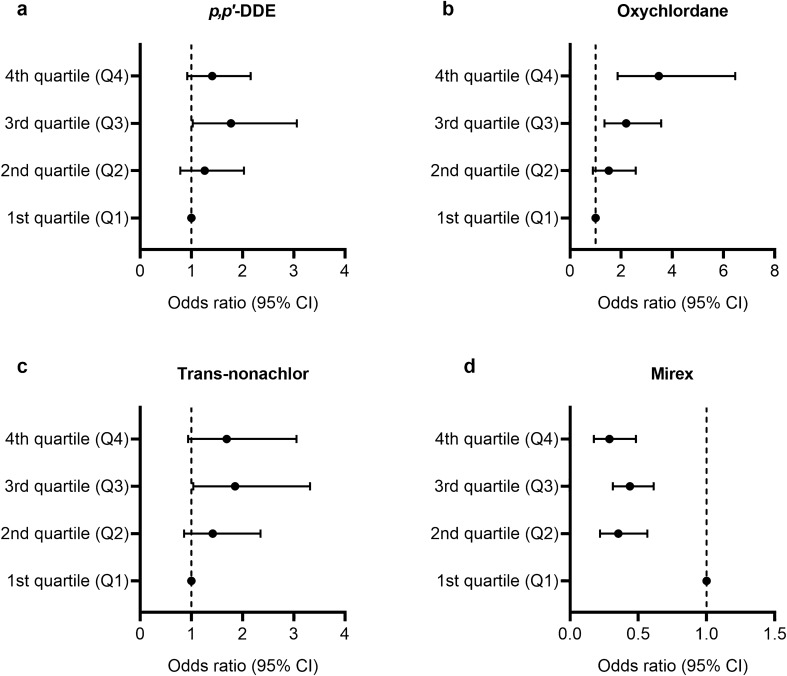
Forest plots of adjusted odds ratio (95% CI) for fatty liver index (≥ 60) according to the exposure quartile for organochlorine pesticides subclasses. (**a**) *p,p′*-DDE, (**b**) Oxychlordane, (**c**) Trans-nonachlor, (**d**) Mirex. The contents of Model 3 in Table 2 are shown as plots. Model 3 was adjusted for age, sex, race, poverty income ratio, smoking, drinking, and physical activity. *CI* confidence interval.

We compared the adjusted ORs for FLI ≥ 60 according to the concentration of each OCP substance (Table 2 and Fig. 2). Only two of the investigated OCP subclasses showed a significant dose-dependent association with the adjusted OR for FLI ≥ 60. Oxychlordane showed a linear relationship with the risk of NAFLD (model 3; OR 3.471 in the fourth quartile [Q4]; 95% CI 1.865–6.458; *P* = 0.007). In contrast, mirex showed a negative correlation with the risk of NAFLD (model 3; OR 0.29 in Q4; 95% CI 0.175–0.483; *P* < 0.001). *p,p′*-DDE and trans-nonachlor were not significantly associated with NAFLD.

**Table 2:** Adjusted odds ratios for fatty liver index (≥ 60) according to the exposure quartiles for organochlorine pesticides subclasses.

	% (SE)	OR (95% CI)		
		Model 1	Model 2	Model 3
***p,p'*-DDE**				
Q1	35.5 (2.65)	1 (ref.)	1 (ref.)	1 (ref.)
Q2	39.9 (3.42)	1.209 (0.78, 1.875)	1.198 (0.747, 1.919)	1.261 (0.784, 2.029)
Q3	50.7 (3.71)	1.869 (1.23, 2.841)	1.699 (1.004, 2.877)	1.774 (1.027, 3.062)
Q4	44.8 (4.08)	1.474 (1.058, 2.054)	1.361 (0.832, 2.227)	1.409 (0.92, 2.157)
*P* value		0.015	0.24	0.24
**Oxychlordane**				
Q1	30.9 (3.09)	1 (ref.)	1 (ref.)	1 (ref.)
Q2	39.7 (3.26)	1.474 (0.912, 2.382)	1.593 (0.944, 2.686)	1.519 (0.899, 2.569)
Q3	46.3 (4.8)	1.93 (1.33, 2.803)	2.191 (1.424, 3.373)	2.196 (1.352, 3.566)
Q4	54.7 (3.12)	2.7 (1.9, 3.839)	3.354 (1.953, 5.759)	3.471 (1.865, 6.458)
*P* value		< 0.001	0.002	0.007
**Trans-nonachlor**				
Q1	31.9 (3.96)	1 (ref.)	1 (ref.)	1 (ref.)
Q2	40.5 (2.18)	1.453 (0.934, 2.26)	1.444 (0.898, 2.322)	1.42 (0.857, 2.352)
Q3	49.7 (2.73)	2.103 (1.302, 3.397)	1.927 (1.099, 3.38)	1.856 (1.039, 3.317)
Q4	48.2 (4.3)	1.984 (1.359, 2.897)	1.653 (0.955, 2.859)	1.693 (0.938, 3.055)
*P* value		0.007	0.16	0.18
**Mirex**				
Q1	54.3 (3.1)	1 (ref.)	1 (ref.)	1 (ref.)
Q2	32.6 (3.31)	0.407 (0.26, 0.636)	0.38 (0.249, 0.582)	0.354 (0.221, 0.567)
Q3	44.4 (3.92)	0.674 (0.474, 0.959)	0.475 (0.333, 0.678)	0.439 (0.314, 0.613)
Q4	36.3 (3.16)	0.479 (0.343, 0.671)	0.289 (0.185, 0.451)	0.29 (0.175, 0.483)
*P* value		0.002	< 0.001	< 0.001

Model 1: Non-adjusted. Model 2: Adjusted for age, sex, race. Model 3: Adjusted for age, sex, race, poverty income ratio, smoking, drinking, physical activity.

Subjects were divided into four categories (Q1 to Q4), ranging from the lowest quartile group to the highest quartile group.

*OR* odds ratio, *CI* confidence interval, *SE* standard error, *ref.* reference group.

Subgroup analyses were performed to determine whether the association between OCP exposure and NAFLD prevalence differed according to sex (Supplementary Table S2) or race (Supplementary Table S3). All four OCPs showed similar patterns in NAFLD prevalence regardless of sex. In contrast, all four OCPs showed significant differences in the association with the prevalence of NAFLD according to race (i.e., Mexican American vs. Other Hispanic, Non-Hispanic White, Non-Hispanic Black, Other Race).

### Correlation between OCP exposure and the constituent variables of the fatty liver index

**Figure. 3:**
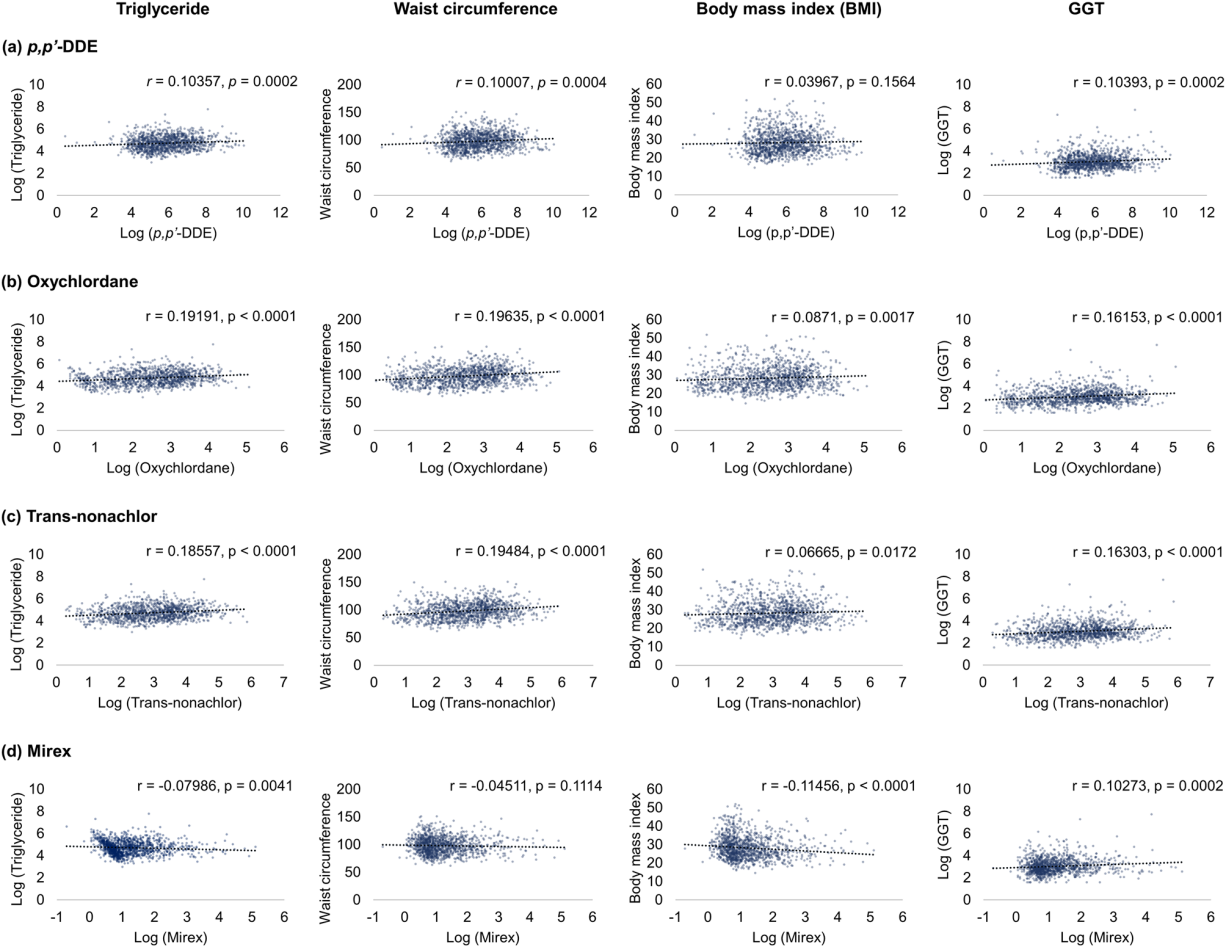
Correlation analysis between log-transformed OCP serum concentrations and FLI component variables. (**a**) *p,p′*-DDE, (**b**) Oxychlordane, (**c**) Trans-nonachlor, (**d**) Mirex. The correlation coefficients (*r*) and *P* values are shown in each scatter plot. *GGT* gamma-glutamyl transferase, *CI* confidence interval.

Each variable constituting FLI was analyzed to examine the correlation with the serum concentration of OCPs. *p,p'*-DDE showed weak positive correlations with triglyceride (*r* = 0.104; *P* < 0.001), waist circumference (*r* = 0.100; *P* < 0.001), and GGT (*r* = 0.104; *P* < 0.001) but had no significant correlation with BMI (*r* = 0.040; *P* = 0.16) (Fig. 3a). Oxychlordane showed weak positive correlations with all FLI parameters, including triglyceride (*r* = 0.192; *P* < 0.001), waist circumference (*r* = 0.196; *P* < 0.001), BMI (*r* = 0.087; *P* = 0.002), and GGT (*r* = 0.162; *P* < 0.001) (Fig. 3b). The results of trans-nonachlor showed a similar trend with oxychlordane (Fig. 3c). On the other hand, mirex showed weak negative correlations with triglyceride (*r* = − 0.080; *P* = 0.004) and BMI (*r* = − 0.115; *P* < 0.001) and a weak positive correlation with GGT (*r* = 0.103; *P* < 0.001) and no significant correlation with waist circumference (*r* = − 0.045; *P* = 0.11) (Fig. 3d).

### Comparison of liver enzyme levels according to the exposure to OCP substance

**Figure. 4:**
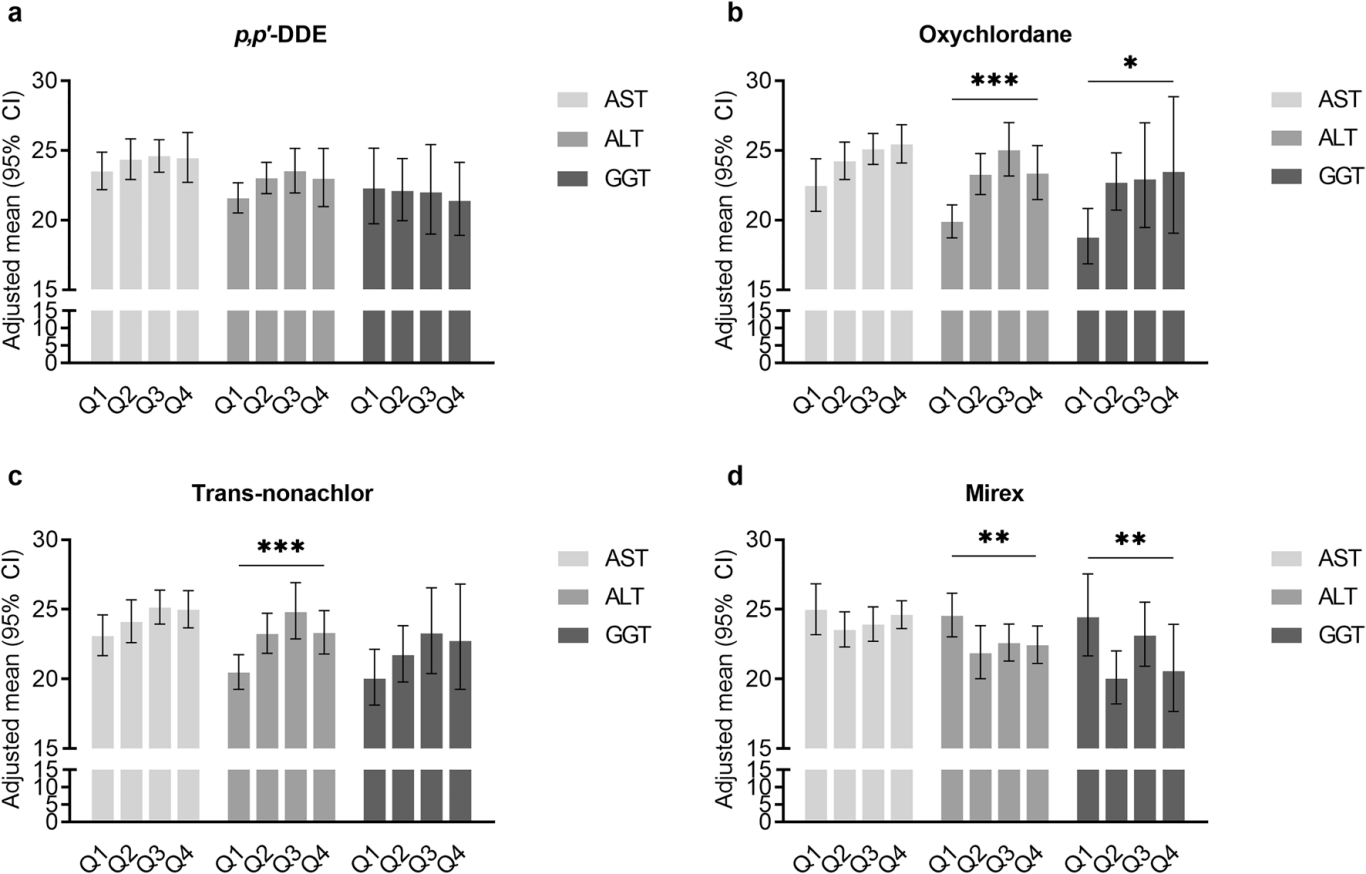
Adjusted means (95% CI) for other NAFLD-related variables (AST, ALT, GGT) according to the quartiles of organochlorine pesticide subclasses. (**a**) *p,p′*-DDE, (**b**) Oxychlordane, (**c**) Trans-nonachlor, (**d**) Mirex. The contents of Model 3 in Table 3 are shown as plots. Model 3 was adjusted for age, sex, race, poverty income ratio, smoking, drinking, and physical activity. *AST* aspartate transaminase, *ALT* alanine transaminase, *GGT* gamma-glutamyl transferase, *CI* confidence interval. *p < 0.05, **p < 0.01, ***p < 0.001.

The adjusted means of liver enzymes were compared according to the degree of exposure to each OCP substance. For *p,p'*-DDE, the adjusted means of all liver enzyme levels did not significantly differ with increasing concentration. For oxychlordane, the adjusted means of ALT and GGT tended to increase according to the quartiles divided by the exposure concentration. For trans-nonachlor, the adjusted mean of ALT was elevated in the high-exposure group, similar to oxychlordane; however, trans-nonachlor did not show a significant difference among the quartiles for the adjusted means of GGT. For mirex, unlike other OCPs, the adjusted means for ALT and GGT tended to decrease in the higher quartiles. All four substances did not show significant differences in the adjusted means for AST level according to quartiles (Table 3 and Fig. 4).

**Table 3:** Adjusted means (95% CI) for other NAFLD-related variables (AST, ALT, GGT) according to the quartiles of organochlorine pesticide subclasses.

	Adjusted Mean (95% CI) (mg/dL)								
	AST			ALT			GGT		
	Model 1	Model 2	Model 3	Model 1	Model 2	Model 3	Model 1	Model 2	Model 3
***p,p'*-DDE (ng/g)**									
Q1	22.67 (21.76–23.62)	23.76 (22.41–25.2)	23.49 (22.19–24.87)	22.2 (21.26–23.17)	21.79 (20.58–23.07)	21.56 (20.51–22.67)	20.27 (18.72–21.95)	22.47 (19.94–25.33)	22.28 (19.74–25.16)
Q2	23.65 (22.65–24.69)	24.51 (23.12–25.98)	24.34 (22.92–25.84)	22.88 (21.64–24.19)	22.67 (21.53–23.87)	22.99 (21.9–24.14)	20.27 (18.69–21.98)	21.79 (19.68–24.12)	22.08 (19.96–24.42)
Q3	23.92 (22.92–24.97)	24.31 (23.15–25.54)	24.58 (23.44–25.77)	23.23 (22.01–24.53)	23.18 (21.59–24.9)	23.5 (21.95–25.15)	22.06 (19.82–24.56)	22.2 (19.31–25.52)	21.98 (19–25.42)
Q4	24.05 (22.87–25.29)	24.38 (23–25.83)	24.43 (22.71–26.29)	21.94 (20.4–23.6)	22.85 (21.1–24.74)	22.96 (20.97–25.15)	21.24 (19.15–23.56)	21.21 (19.14–23.49)	21.37 (18.91–24.15)
*P* value	0.17	0.61	0.34	0.47	0.35	0.08	0.47	0.76	0.93
**Oxychlordane (ng/g)**									
Q1	22.27 (21.13–23.47)	22.74 (20.9–24.74)	22.44 (20.64–24.4)	21.23 (20.03–22.51)	20.15 (18.76–21.64)	19.87 (18.72–21.09)	17.75 (16.24–19.41)	18.8 (16.68–21.18)	18.74 (16.86–20.83)
Q2	23.64 (22.89–24.41)	24.53 (23.19–25.94)	24.22 (22.91–25.6)	23.61 (22.63–24.64)	23.51 (22.08–25.03)	23.26 (21.83–24.77)	21.62 (19.98–23.38)	23.23 (21.07–25.6)	22.67 (20.71–24.82)
Q3	24.02 (23–25.09)	24.93 (23.85–26.05)	25.08 (23.99–26.22)	23.72 (21.99–25.58)	24.43 (22.59–26.42)	25.01 (23.16–27)	21.81 (19.11–24.88)	22.67 (19.3–26.64)	22.92 (19.46–26.98)
Q4	24 (23.04–25)	24.98 (23.74–26.3)	25.43 (24.09–26.85)	21.04 (19.43–22.8)	22.78 (21.09–24.61)	23.33 (21.48–25.34)	22.45 (19.55–25.77)	22.94 (19.05–27.63)	23.46 (19.06–28.86)
*P* value	0.040	0.13	0.09	0.010	< 0.001	< 0.001	0.009	0.034	0.022
**Trans-nonachlor (ng/g)**									
Q1	22.67 (21.67–23.71)	23.39 (21.73–25.19)	23.09 (21.68–24.61)	21.62 (20.42–22.9)	20.78 (19.21–22.48)	20.47 (19.27–21.75)	18.66 (17.13–20.33)	20.33 (18.28–22.61)	20.03 (18.14–22.13)
Q2	23.08 (22.18–24.02)	24.13 (22.68–25.67)	24.09 (22.61–25.68)	22.76 (21.77–23.79)	23 (21.52–24.58)	23.24 (21.85–24.72)	20.36 (18.79–22.07)	22.09 (20.07–24.32)	21.72 (19.79–23.83)
Q3	24.4 (23.57–25.24)	25.1 (24.08–26.18)	25.13 (23.94–26.37)	24.06 (22.59–25.63)	24.6 (22.84–26.49)	24.81 (22.88–26.91)	22.38 (20.5–24.43)	22.92 (20.15–26.07)	23.27 (20.4–26.55)
Q4	24.08 (23.1–25.11)	24.56 (23.33–25.86)	24.97 (23.67–26.34)	21.7 (20.31–23.18)	22.64 (21.37–24)	23.3 (21.8–24.9)	22.83 (20.83–25.03)	22.17 (19.2–25.6)	22.73 (19.26–26.82)
*P* value	0.006	0.34	0.10	0.044	0.022	< 0.001	0.010	0.48	0.23
**Mirex (ng/g)**									
Q1	23.74 (22.73–24.8)	24.87 (23.21–26.66)	24.95 (23.19–26.83)	23.87 (22.81–24.99)	24.43 (22.75–26.24)	24.54 (23.03–26.15)	21.45 (20.29–22.66)	24.17 (21.82–26.78)	24.43 (21.67–27.54)
Q2	22.39 (21.92–22.87)	23.44 (22.22–24.72)	23.53 (22.31–24.82)	21.25 (20.11–22.45)	21.62 (19.85–23.55)	21.85 (20.03–23.83)	17.9 (16.81–19.07)	19.84 (18.17–21.66)	20.03 (18.22–22.02)
Q3	23.41 (22.42–24.44)	23.75 (22.6–24.96)	23.91 (22.71–25.17)	22.73 (21.42–24.13)	22.3 (21.01–23.68)	22.58 (21.3–23.94)	23.15 (21.49–24.94)	23.3 (21.28–25.51)	23.11 (20.92–25.52)
Q4	24.7 (23.93–25.49)	24.85 (23.95–25.79)	24.6 (23.63–25.61)	22.64 (21.36–24)	22.34 (21.12–23.63)	22.43 (21.13–23.81)	21.51 (18.71–24.73)	20.6 (17.83–23.81)	20.56 (17.67–23.93)
*P* value	< 0.001	0.10	0.19	0.007	0.006	0.003	< 0.001	< 0.001	0.001

Model 1: Non-adjusted. Model 2: Adjusted for age, sex, race. Model 3: Adjusted for model 2 and poverty income ratio, smoking, drinking, physical activity.

Subjects were divided into four categories (Q1 to Q4), ranging from the lowest quartile group to the highest quartile group.

*AST* aspartate transaminase, *ALT* alanine transaminase, *GGT* gamma-glutamyl transferase, *CI* confidence interval.

### Association between OCP exposure and advanced hepatic fibrosis

We also assessed whether the risk of developing advanced hepatic fibrosis differed according to the concentration of OCPs. When advanced hepatic fibrosis was defined as FIB-4 of 2.67 or higher, there were no significant associations between the concentrations of all four substances with the risk of advanced hepatic fibrosis (Supplementary Table S4).

## Discussion

In this study based on a nationwide health and nutrition survey in the United States, we found that the presence of NAFLD as defined by FLI ≥ 60 was significantly associated with the degree of OCP exposure in adults. A significant association was found for some OCP, such as oxychlordane. The adjusted OR of NAFLD was significantly higher at high concentrations of serum oxychlordane. Considering that oxychlordane has a higher toxicity and bioaccumulation potential than trans-nonachlor^21,22^, it could be inferred that the dose-dependency of oxychlordane in NAFLD prevalence might have been attributed to its high bioaccumulation properties. Similarly, *p,p′-*DDE and trans-nonachlor showed an increased serum concentration relevant to NAFLD. Previous studies showed that *p,p′*-DDE was associated with increases in BMI, triglycerides, insulin resistance, and reductions in HDL cholesterol^7,23^. In addition, trans-nonachlor has a pro-steatotic effect on hepatocytes in animal experiments^24^. Our study suggests that the prevalence of NAFLD may have an independent association with the concentration of OCPs in the general population, some of which showed a linear dose-dependent relationship.

Several studies have reported that the concentrations of OCP substances differed according to sex^9,25,26^; however, in terms of the prevalence of NAFLD according to OCP exposure, previous studies did not observe a significant difference according to sex^15^, which is consistent with our results. In contrast, OCP exposure and the prevalence of NAFLD were shown to be different according to race^9,25,26^, which is often associated with differences in other living-environmental factors such as dietary habits, occupations, and socioeconomic status, or genetic susceptibility to OCP exposure. Further research is needed to determine the factors and mechanisms involved in the sex and ethnic difference in NAFLD prevalence according to OCP exposure.

Overall, the tendency for a higher concentration of OCPs was correlated with the presence of hypertriglyceridemia and obesity. Because OCPs are lipophilic, individuals with high BMI will accumulate more OCPs in the body than those with low BMI when exposed to the same amount of pesticides^27^. However, few epidemiologic studies previously established an association between OCPs and obesity or hypertriglyceridemia^28^. Lee et al. found that among various OCPs, only *p,p'*-DDE was positively associated with the adjusted means of BMI, and *p,p'*-DDE, oxychlordane, and trans-nonachlor were positively correlated with the adjusted triglyceride levels^7^. Our results could be explained in a similar aspect to previous studies; however, further studies are needed to determine whether metabolic dysregulation significantly accelerates the development of obesity and hepatic steatosis by OCP exposure.

We found that the adjusted values of overall liver enzymes in most OCPs tended to increase according to OCP exposure. These results firmly supported the evidence for the relationship between OCPs and liver enzyme elevation shown in previous studies^4,15,29^. The reason why the trends observed in FLI were similar to the adjusted means of ALT and GGT according to the serum concentrations of oxychlordane and mirex may be that ALT and GGT have been used as conventional markers of NAFLD. When compared with the most recent cutoff values of ALT (> 30 IU/L for men and > 19 IU/L for women) for NAFLD prediction^30^, the adjusted mean levels of ALT of both low and high OCP concentrations (i.e., Q1 and Q4) were not much higher than the cutoff level. In addition, when compared with the cutoff value of GGT (< 50 IU/L) as an indicator of liver dysfunction^31,32^, the adjusted mean levels of GGT of low and high OCP concentrations (Q1 and Q4) were both within the cutoff value. In real-world clinical practice, it may be challenging to determine the presence of NAFLD only by using an elevation of liver enzymes in OCP-exposed patients.

Our study results showed no significant difference in the prevalence of advanced hepatic fibrosis according to the concentration of all four OCP substances. One possible explanation for this finding is that FIB-4 did not reflect actual hepatic fibrosis in our study setting. In general, serologic markers cannot adequately detect toxicant-associated steatohepatitis, even with advanced hepatic fibrosis in liver biopsy^33^. Another explanation is that the exposure to OCPs was not sufficiently long for advanced fibrosis to occur. Therefore, further research is needed to determine whether advanced hepatic fibrosis depends on the degree of OCP exposure.

This is the first study to use the FLI to analyze the association between OCPs and NAFLD. Among the serologic markers of NAFLD, FLI has been repeatedly validated and widely used in epidemiologic studies and has good discriminatory power for NAFLD because it is not affected by transaminase levels, while ALT can be altered by the presence of viral hepatitis or alcoholic hepatitis^34^. FLI can also avoid the failure for NAFLD classification associated with normal transaminase levels^18,34^. Contrary to our study, a previous NHANES data study did not exclude patients with viral hepatitis and heavy drinkers from the analysis and only adjusted for age and sex^29^. Since the effects of viral hepatitis and heavy alcoholics on liver enzyme elevation were excluded in our study, our results may be more reliable for examining elevations in liver enzymes that are solely due to NAFLD.

Interestingly, our study showed a notable result concerning mirex, which showed an opposite tendency with oxychlordane in terms of the prevalence of NAFLD. Previous animal studies reported that chlordecone, mirex, and chlordane trigger chemical-induced steatosis^35^. Interestingly, the prevalence of metabolic syndrome was higher at lower concentrations of mirex in previous studies^9^, which is similar to the trends in the prevalence of NAFLD for mirex shown in this study. As such, the common features found in the association between mirex and other metabolic diseases including NAFLD do not seem to be in line with the results of existing animal studies. Furthermore, the association between the duration of OCP exposure or concentration difference with steatosis had not been analyzable. Therefore, it may be difficult to determine whether all classes of OCPs have a concentration-dependent tendency to induce hepatic steatosis.

The proposed mechanism of the induction of NAFLD by OCPs is the induction of oxidative stress by increasing reactive oxygen species production via the activation of cytochrome P450 expression, which is related to the detoxification pathway in the liver, or by affecting lipid metabolism^36–38^. Animal studies and population-based studies have reported that OCP exposure was associated with DNA hypomethylation^39,40^, which in turn is related to the occurrence of NAFLD^41^. Further studies are needed to elucidate whether such a mechanism can explain the association between OCPs and NAFLD and the effect of differences in the chemical properties of each OCP on the prevalence of NAFLD.

This study has limitations in verifying the causality over time due to the nature of its cross-sectional design. Because it is uncertain whether ultrasound or biopsy was performed, participants who did not have NAFLD might have been included in the results. The use of a serum-based scoring system to define NAFLD and advanced fibrosis inevitably has accuracy issues. Although liver ultrasound transient elastography (FibroScan®) data is now widely used to evaluate NAFLD and advanced fibrosis, FibroScan® was introduced in 2017 and its data were not available in the NHANES 2003–2004 database. It was also impossible to analyze the association between OCP exposure and NAFLD prevalence in the recent NHANES data from 2005 to 2016 because the pooled-sample data file of OCPs could not be linked to other NHANES data, including the variables of FLI. Also, this study did not separately consider the effects of simultaneous exposure to different types of OCPs and multiple kinds of other EDCs on NAFLD. In addition, it was not possible to directly determine whether the presence of NAFLD is related to environmental effects such as complex OCP exposure history, region, and diet, or through the precedent of metabolic syndrome. Further studies are needed to clarify the above-mentioned points.

## Conclusions

Our results showed that OCP exposure was associated with NAFLD prevalence, some of which showed a linear dose-dependent relationship. Although most pesticides have been deprecated, periodic monitoring for NAFLD appears necessary in developing countries where pesticides are still used or in areas in which pesticides have been used in the past. Further studies using in vivo experiments are needed to clarify the mechanism of the influence of OCPs on the pathogenesis of NAFLD.

## Supplementary Information

Supplementary Tables.

## Data Availability

The datasets used during the current study are available from the National Health and Nutrition Examination Survey (NHANES). The data can be accessed from the website: https://wwwn.cdc.gov/nchs/nhanes/2003-2004/L28OCP_C.htm.

## Abbreviations

EDC: Endocrine disrupting chemical
OCP: Organochlorine pesticide
NAFLD: Nonalcoholic fatty liver disease
AST: Aspartate transaminase
ALT: Alanine transaminase
GGT: Gamma-glutamyl transferase
FLI: Fatty liver index
NHANES: National Health and Nutrition Examination Survey
IRB: Institutional Review Board
DDE: Dichlorodiphenyldichloroethylene
FIB-4: Fibrosis-4 index
OR: Odds ratio
CI: Confidence interval
BMI: Body mass index
