# Supplementary material for: Association between organochlorine pesticides and nonalcoholic fatty liver disease in the National Health and Nutrition Examination Survey 2003–2004
Source: Sci Rep. 2022 Jul 8;12:11590. doi: 10.1038/s41598-022-15741-2 (PMC9270488; doi:10.1038/s41598-022-15741-2)
Supplement: Supplementary file 1 — Supplementary Tables. [file 41598_2022_15741_MOESM1_ESM.docx]

**Association between Organochlorine Pesticides and Nonalcoholic Fatty Liver Disease in the National Health and Nutrition Examination Survey 2003–2004**

**Running title**: Organochlorine Pesticides and Nonalcoholic Fatty Liver Disease

Hyunji Sang^1^, Kyu-Na Lee^2^, Chang Hee Jung^1^, Kyungdo Han^3^, Eun Hee Koh^1, *^

^1^Department of Internal Medicine, Asan Medical Center, University of Ulsan College of Medicine, Seoul, Republic of Korea. ^2^Department of Biomedicine & Health Science, The Catholic University of Korea, Seoul, Republic of Korea. ^3^Department of Statistics and Actuarial Science, Soongsil University, Seoul, Republic of Korea. **^*^** e-mail: ehk@amc.seoul.kr

**Supplementary Table S1**. Cutoff values according to the quartiles of organochlorine pesticide subclasses

| **Quartile** | **Cutoff value (ng/g)** | | | |
| --- | --- | --- | --- | --- |
|  | *p,p’*-DDE | Oxychlordane | Trans-nonachlor | Mirex |
| Q1 | ≤ 138 | ≤ 6 | ≤ 9.4 | ≤ 2 |
| Q2 | ≤ 329 | ≤ 13 | ≤ 20.2 | ≤ 2.76 |
| Q3 | ≤ 875 | ≤ 24.5 | ≤ 38.2 | ≤ 5 |
| Q4 | > 875 | > 24.5 | > 38.2 | > 5 |

Subjects were divided into four categories (Q1 to Q4), ranging from the lowest quartile group to the highest quartile group.

Q1, first quartile (≤ 25^th^ percentile); Q2, second quartile (25^th^–50^th^ percentile); Q3, third quartile (50^th^–75^th^ percentile); Q4, fourth quartile (> 75^th^ percentile).

**Supplementary Table S2.** Adjusted odds ratios for fatty liver index (≥ 60) according to the exposure quartiles for organochlorine pesticides subclasses: subgroup analysis by sex

|  |  | % (SE) | Adjusted OR (95% CI) |
| --- | --- | --- | --- |
| ***p,p'*-DDE** | |  | |
| Male | Q1 | 43.9 (3.92) | 1 (ref.) |
|  | Q2 | 52.22 (4.66) | 1.426 (0.763, 2.665) |
|  | Q3 | 58.88 (4.2) | 1.672 (0.855, 3.269) |
|  | Q4 | 58.46 (6.31) | 1.371 (0.614, 3.063) |
| Female | Q1 | 27.3 (3.39) | 1 (ref.) |
|  | Q2 | 29.09 (3.59) | 1.167 (0.59, 2.31) |
|  | Q3 | 42.6 (5.87) | 1.868 (0.865, 4.035) |
|  | Q4 | 36.02 (4.52) | 1.432 (0.755, 2.716) |
| *P* value for interaction | |  | 0.826 |
| **Oxychlordane** | |  |  |
| Male | Q1 | 38.12 (4.05) | 1 (ref.) |
|  | Q2 | 52.87 (4.44) | 1.536 (0.772, 3.054) |
|  | Q3 | 53.75 (6.66) | 1.657 (0.88, 3.12) |
|  | Q4 | 73.4 (5.6) | 4.208 (1.987, 8.913) |
| Female | Q1 | 23.71 (2.8) | 1 (ref.) |
|  | Q2 | 28.09 (4.58) | 1.483 (0.822, 2.676) |
|  | Q3 | 39.95 (4.6) | 2.713 (1.593, 4.622) |
|  | Q4 | 40.51 (4.57) | 3.096 (1.285, 7.458) |
| *P* value for interaction | |  | 0.227 |
| **Trans-nonachlor** | |  |  |
| Male | Q1 | 40.33 (6.18) | 1 (ref.) |
|  | Q2 | 50.89 (3.85) | 1.281 (0.556, 2.949) |
|  | Q3 | 62.64 (5.04) | 1.848 (0.745, 4.584) |
|  | Q4 | 56.81 (4.99) | 1.391 (0.766, 2.525) |
| Female | Q1 | 24.34 (2.73) | 1 (ref.) |
|  | Q2 | 32.5 (3.65) | 1.555 (0.941, 2.572) |
|  | Q3 | 37.81 (4.26) | 1.82 (0.928, 3.567) |
|  | Q4 | 39.12 (5.19) | 1.997 (0.759, 5.254) |
| *P* value for interaction | |  | 0.665 |
| **Mirex** | |  |  |
| Male | Q1 | 72.09 (4.2) | 1 (ref.) |
|  | Q2 | 38.6 (5.69) | 0.204 (0.083, 0.501) |
|  | Q3 | 56.55 (5.24) | 0.335 (0.163, 0.687) |
|  | Q4 | 43.78 (4.14) | 0.207 (0.069, 0.621) |
| Female | Q1 | 43.75 (3) | 1 (ref.) |
|  | Q2 | 28.39 (3.13) | 0.468 (0.322, 0.679) |
|  | Q3 | 29.7 (4.15) | 0.449 (0.272, 0.742) |
|  | Q4 | 26.22 (5.02) | 0.302 (0.127, 0.719) |
| *P* value for interaction | |  | 0.184 |

The adjusted ORs were based on Model 3 (adjusted for age, sex, race, poverty income ratio, smoking, drinking, and physical activity) described in Table 2.

“*P* value for interaction” refers to the significance between sexes and was calculated through logistic regression analysis.

Subjects were divided into four categories (Q1 to Q4), ranging from the lowest quartile group to the highest quartile group.

*OR* odds ratio, *CI* confidence interval, *SE* standard error, *ref.* reference group.

**Supplementary Table S3.** Adjusted odds ratios for fatty liver index (≥ 60) according to the exposure quartiles for organochlorine pesticides subclasses: subgroup analysis by race

|  |  | % (SE) | Adjusted OR (95% CI) |
| --- | --- | --- | --- |
| ***p,p'*-DDE** | | |  |
| Mexican American | Q1 | 40.64 (10.44) | 1 (ref.) |
|  | Q2 | 40.1 (6.12) | 0.978 (0.285, 3.359) |
|  | Q3 | 48.08 (6.74) | 1.353 (0.543, 3.372) |
|  | Q4 | 42.43 (7.5) | 1.077 (0.396, 2.926) |
| Other Hispanic | Q1 | 32.12 (21.89) | 1 (ref.) |
|  | Q2 | 35.71 (15.15) | 1.174 (0.1, 13.81) |
|  | Q3 | 55.49 (22.99) | 2.635 (0.807, 8.607) |
|  | Q4 | 40.31 (20.29) | 1.427 (0.065, 31.405) |
| Non-Hispanic White | Q1 | 35.56 (2.43) | 1 (ref.) |
|  | Q2 | 40.33 (4.56) | 1.225 (0.725, 2.07) |
|  | Q3 | 53.85 (4.57) | 2.114 (1.3, 3.44) |
|  | Q4 | 47.22 (5.35) | 1.622 (1.092, 2.408) |
| Non-Hispanic Black | Q1 | 34.93 (6.81) | 1 (ref.) |
|  | Q2 | 34.84 (6.65) | 0.996 (0.394, 2.517) |
|  | Q3 | 52.11 (8.1) | 2.027 (0.955, 4.301) |
|  | Q4 | 53.17 (4.76) | 2.115 (0.945, 4.736) |
| Other Race | Q1 | 33.44 (18.37) | 1 (ref.) |
|  | Q2 | 44.36 (10.83) | 1.587 (0.229, 10.977) |
|  | Q3 | 13.14 (11.72) | 0.301 (0.019, 4.867) |
|  | Q4 | 16.62 (12.78) | 0.397 (0.023, 6.897) |
| *P* value for interaction | |  | 0.007 |
| **Oxychlordane** | | |  |
| Mexican American | Q1 | 37.31 (5.13) | 1 (ref.) |
|  | Q2 | 49.2 (5.62) | 1.627 (0.92, 2.876) |
|  | Q3 | 45.25 (9.11) | 1.389 (0.621, 3.104) |
|  | Q4 | 56 (4.34) | 2.138 (1.068, 4.28) |
| Other Hispanic | Q1 | 16.9 (9.03) | 1 (ref.) |
|  | Q2 | 50.17 (17.48) | 4.951 (0.54, 45.42) |
|  | Q3 | 63.79 (16.86) | 8.663 (1.062, 70.693) |
|  | Q4 | 34.39 (29.54) | 2.577 (0.049, 135.335) |
| Non-Hispanic White | Q1 | 31.08 (4.31) | 1 (ref.) |
|  | Q2 | 40.88 (3.49) | 1.533 (0.808, 2.909) |
|  | Q3 | 45.93 (5.92) | 1.883 (1.135, 3.123) |
|  | Q4 | 53.48 (3.23) | 2.549 (1.668, 3.895) |
| Non-Hispanic Black | Q1 | 31.41 (5.64) | 1 (ref.) |
|  | Q2 | 29.55 (6.74) | 0.916 (0.412, 2.037) |
|  | Q3 | 53.1 (7.1) | 2.472 (1.103, 5.539) |
|  | Q4 | 65.56 (5.26) | 4.157 (2.474, 6.985) |
| Other Race | Q1 | 24.32 (8.83) | 1 (ref.) |
|  | Q2 | 18.32 (8.23) | 0.698 (0.168, 2.9) |
|  | Q3 | 25.12 (16.67) | 1.044 (0.168, 6.479) |
|  | Q4 | 58.86 (18.24) | 4.452 (0.495, 40.054) |
| *P* value for interaction | |  | < 0.001 |
| **Trans-nonachlor** | | |  |
| Mexican American | Q1 | 41.99 (4.82) | 1 (ref.) |
|  | Q2 | 36.93 (6.97) | 0.809 (0.369, 1.772) |
|  | Q3 | 52.51 (8.27) | 1.528 (0.781, 2.987) |
|  | Q4 | 50.36 (8.18) | 1.402 (0.67, 2.933) |
| Other Hispanic | Q1 | 30.03 (12.05) | 1 (ref.) |
|  | Q2 | 12.48 (8.59) | 0.332 (0.094, 1.177) |
|  | Q3 | 62.04 (18.38) | 3.809 (0.386, 37.607) |
|  | Q4 | 82.74 (17.7) | 11.166 (0.579, 215.155) |
| Non-Hispanic White | Q1 | 31.65 (5.6) | 1 (ref.) |
|  | Q2 | 42.81 (2.88) | 1.617 (0.833, 3.138) |
|  | Q3 | 49.65 (3.73) | 2.13 (1.073, 4.226) |
|  | Q4 | 45.68 (5.18) | 1.816 (1.044, 3.16) |
| Non-Hispanic Black | Q1 | 27.54 (3.75) | 1 (ref.) |
|  | Q2 | 38.34 (8.85) | 1.637 (0.644, 4.156) |
|  | Q3 | 44.68 (7.41) | 2.125 (1.228, 3.678) |
|  | Q4 | 66.18 (5.97) | 5.149 (2.424, 10.938) |
| Other Race | Q1 | 24.25 (9.6) | 1 (ref.) |
|  | Q2 | 21.87 (10.71) | 0.874 (0.156, 4.91) |
|  | Q3 | 43.93 (15.09) | 2.448 (0.532, 11.27) |
|  | Q4 | 28.12 (22.79) | 1.222 (0.058, 25.699) |
| *P* value for interaction | |  | < 0.001 |
| **Mirex** | | |  |
| Mexican American | Q1 | 60.37 (6.16) | 1 (ref.) |
|  | Q2 | 32.66 (8.12) | 0.318 (0.094, 1.083) |
|  | Q3 | 36.67 (6.65) | 0.38 (0.157, 0.919) |
|  | Q4 | 26.78 (10.71) | 0.24 (0.064, 0.901) |
| Other Hispanic | Q1 | 51.71 (16.88) | 1 (ref.) |
|  | Q2 | 30.78 (21.18) | 0.415 (0.036, 4.834) |
|  | Q3 | 32.01 (17.25) | 0.44 (0.035, 5.547) |
|  | Q4 | 40.23 (25.49) | 0.629 (0.123, 3.201) |
| Non-Hispanic White | Q1 | 55.54 (3.95) | 1 (ref.) |
|  | Q2 | 31.08 (3.65) | 0.361 (0.21, 0.622) |
|  | Q3 | 47.25 (4.09) | 0.717 (0.459, 1.121) |
|  | Q4 | 36.11 (4.68) | 0.452 (0.289, 0.709) |
| Non-Hispanic Black | Q1 | 53.42 (12.19) | 1 (ref.) |
|  | Q2 | 38.72 (6.12) | 0.551 (0.162, 1.875) |
|  | Q3 | 35.88 (10.6) | 0.488 (0.148, 1.606) |
|  | Q4 | 44.51 (6.58) | 0.699 (0.203, 2.41) |
| Other Race | Q1 | 23.55 (13.83) | 1 (ref.) |
|  | Q2 | 39.63 (14.79) | 2.131 (0.268, 16.94) |
|  | Q3 | 30.87 (10.9) | 1.45 (0.15, 13.975) |
|  | Q4 | 9.65 (7.36) | 0.347 (0.027, 4.374) |
| *P* value for interaction | |  | < 0.001 |

The adjusted ORs were based on Model 3 (adjusted for age, sex, race, poverty income ratio, smoking, drinking, and physical activity) described in Table 2.

“*P* value for interaction” refers to the significance among races and was calculated through logistic regression analysis.

Subjects were divided into four categories (Q1 to Q4), ranging from the lowest quartile group to the highest quartile group.

*OR* odds ratio, *CI* confidence interval, *SE* standard error, *ref.* reference group.

**Supplementary Table S4**. Adjusted odds ratios for fibrosis-4 (FIB-4) index ≥ 2.67 according to the exposure quartiles for organochlorine pesticides subclasses

|  |  | % (SE) | OR (95% CI) | | |
| --- | --- | --- | --- | --- | --- |
|  |  |  | Model 1 | Model 2 | Model 3 |
| *p,p'*-DDE | Q1 | 1.56 (0.67) | 1 (ref.) | 1 (ref.) | 1 (ref.) |
|  | Q2 | 1.17 (0.67) | 0.748 (0.152, 3.678) | 0.568 (0.112, 2.875) | 0.125 (0.015, 1.022) |
|  | Q3 | 1.57 (0.68) | 1.008 (0.229, 4.435) | 0.3 (0.07, 1.297) | 0.215 (0.054, 0.853) |
|  | Q4 | 5.07 (1.31) | 3.371 (1.16, 9.795) | 0.583 (0.182, 1.86) | 0.31 (0.082, 1.17) |
|  | *P* value |  | 0.07 | 0.43 | 0.19 |
| Oxychlordane | Q1 | 1.00 (0.79) | 1 (ref.) | 1 (ref.) | 1 (ref.) |
|  | Q2 | 1.29 (0.68) | 1.294 (0.144, 11.65) | 0.628 (0.056, 7.023) | 0.265 (0.011, 6.419) |
|  | Q3 | 1.14 (0.49) | 1.141 (0.149, 8.735) | 0.189 (0.021, 1.673) | 0.153 (0.01, 2.304) |
|  | Q4 | 5.79 (1.04) | 6.076 (1.016, 36.35) | 0.401 (0.055, 2.939) | 0.328 (0.026, 4.17) |
|  | *P* value |  | 0.006 | 0.32 | 0.46 |
| Trans-nonachlor | Q1 | 1.20 (0.81) | 1 (ref.) | 1 (ref.) | 1 (ref.) |
|  | Q2 | 0.89 (0.6) | 0.738 (0.082, 6.643) | 0.233 (0.022, 2.511) | 0.042 (0.001, 1.41) |
|  | Q3 | 1.75 (0.44) | 1.457 (0.279, 7.611) | 0.166 (0.036, 0.767) | 0.075 (0.004, 1.388) |
|  | Q4 | 5.47 (0.81) | 4.743 (1.069, 21.037) | 0.238 (0.045, 1.251) | 0.112 (0.006, 1.956) |
|  | *P* value |  | 0.002 | 0.16 | 0.33 |
| Mirex | Q1 | 1.57 (0.79) | 1 (ref.) | 1 (ref.) | 1 (ref.) |
|  | Q2 | 0.62 (0.32) | 0.389 (0.082, 1.859) | 0.393 (0.078, 1.994) | 0.581 (0.09, 3.743) |
|  | Q3 | 2.52 (0.8) | 1.617 (0.429, 6.095) | 0.814 (0.226, 2.941) | 0.978 (0.242, 3.952) |
|  | Q4 | 3.79 (1.26) | 2.47 (0.604, 10.102) | 0.949 (0.261, 3.45) | 0.813 (0.2, 3.299) |
|  | *P* value |  | 0.10 | 0.60 | 0.90 |

Model 1: Non-adjusted. Model 2: Adjusted for age, sex, race. Model 3: Adjusted for age, sex, race, poverty income ratio, smoking, drinking, physical activity.

Subjects were divided into four categories (Q1 to Q4), ranging from the lowest quartile group to the highest quartile group.

*OR* odds ratio, *CI* confidence interval, *SE* standard error, *ref.* reference group.
